# Remarks on Abstinence; on Insufficient Nourishment; and Their Dangers

**Published:** 1831-05

**Authors:** P. A. Piorry


### ABSTINENCE.

*Remarks on Abstinence; on insufficient Nourishment; and their Dangers.* By P. A. Piorry.

(Concluded from page 303.)

2d. *On the dangers of abstinence in diseases of the lungs.* In acute inflammations of the lungs, especially in young subjects, and when the symptoms are well marked, and the blood is buffed, abstinence is indispensable: it remedies the plasticity of the blood which traverses the lungs, diminishes their volume, and, in concurrence with bloodletting, may tend to promote resolution of the inflammatory action. But it may be doubted whether the same reasoning will apply to those cases of pulmonic congestion which evidently arise from mechanical causes, from want of venous action, or to those states of venous blood which take place in the pulmonic vessels in consequence of disease of the heart; or in old subjects, or those affected by chronic maladies. To diminish quickly the quantity of blood contained in the heart and lungs, and to endeavour to stimulate these organs, in order to give them energy, are, in old persons affected with pneumonia, as the consequence of some mechanical obstruction to the circulation, the two leading indications which present themselves. Whatever may be asserted, it certainly is not *irritation* which then fixes the blood in the lungs, which retains the blood in the most depending part, or which keeps this pretended inflammation so under the laws of gravity, that the points where the disease ceases, and those where the healthy tissue is found, are disposed in a parallel line. It is the weight of the blood which produces this phenomenon, the slowness of the circulation, the weakness of the motive powers, the want of energy in the vessels; in one word, it is the weakness of the solids which renders them incapable of moving the mass of blood. Take away blood, for the purpose of endeavouring to proportion the strength of the organs to the quantity of the fluids: but, so far from putting the patient upon a system of rigorous abstinence, give stimuli, substantial food in small quantities at a time, and, in some cases, diluted alcohol. It is not without very mature reflection that, in such cases, I have employed the mode of treatment I recommend.* But the theory rests upon facts of still greater weight: I found, in the lungs of weak and old persons, who had been debilitated by long-standing organic diseases, large quantities of blood: this engorgement always existed at the most depending part. During life, the contractions of the heart were very feeble; and, after death, the heart was evidently much softened. The lungs, having lost their consistence, appeared as if they had been macerated by the fluids which penetrated them, and during life there was not always that buffy coat upon the blood which is detected in cases of pure pneumonia.

Some patients, labouring under pneumonia, recovered, although they had committed various irregularities in their diet, and had taken soup and wine: many perished who had been kept rigidly abstinent. Numerous cases of pneumonia, united with a morbid condition of the heart, were treated, amongst the old patients in the infirmary of the Salpêtrière, by bleedings; but on the next, or even the same day, soup, light animal food, and even wine, were administered: and I may affirm, from much experience in this mode of treatment, that neither the food nor the wine ever did any harm, and that those pneumonic subjects who were supported by nourishment recovered more favorably, and much more quickly, than those who were limited to a strict and very sparing diet. I foresee the objection, that the transmitted chyle will excite the pulmonic vessels; and I am not unwilling to admit the hypothesis: but I confess I am not inclined to fear this excitation of the vessels of the lungs in an old and weakened

* By referring to Mr. Rennie's "Observations on Asthma, &c." or to the review of his work in our number for December 1830, p. 525, the reader will find that Mr. R. gives a practical exposition of very similar doctrines to those advocated by M. Piorry.—Editor.

subject. It may also be said, that the stomach will have a large quantity of the fluids determined to it, and that thus excessive excitement of the heart will arise: but this excitement is not always to be feared; and often, indeed, it is desirable to rouse the left ventricle, when it is necessary to free the lungs from engorgement of blood; and, admitting a determination of the vital fluids to the stomach, I perceive in this fact a more useful derivation than the painful stimulus which arises from sinapisms, or blisters applied to the thighs.

The regimen in pulmonary consumption merits our most serious attention. Softened tubercles cannot be cured by abstinence; crude tubercles cannot be absorbed by sparing diet; the sputa will not be more easily expectorated by depriving the patient of food. The dreadful effects of the absorption of the puriform matter, which is altered by the air, cannot be prevented by the void which occurs from insufficient nourishment. The partial inflammation which surrounds pulmonary tubercles will not be arrested by refusing to satisfy the appetite of the patient. Thus, abstinence from food, by deteriorating the quality of the blood, and favoring dangerous absorptions, by not mingling the putrid matters which are absorbed with healthy chyle, which may counterbalance their serious effects, by diminishing the general powers of the patient, and irritating the stomach, will remedy no single symptom, and will add an additional evil to the already lamentable disease. Few modern physicians, indeed, propose rigorous abstinence to phthisical patients; but they, in general, allow only vegetable diet, which is insufficient, and sometimes pernicious. Pathological anatomy teaches us that tuberculous ulcerations of the intestines, are the most frequent causes of the sinking and death of consumptive patients. These ulcerations are commonly very distant from the stomach. On the other hand, experiments upon living animals, and some cases of artificial anus in man, prove that vegetable substances, incompletely digested in the stomach, which they rapidly pass through, remain for a long time in the intestines, and produce irritation in them. Is it, therefore, proper to deprive a consumptive patient of animal food, when the symptoms shew that the stomach is healthy? and to give vegetable nourishment to such patients, when we know that herbivorous animals are most subject to tubercles? or to refuse them animal food, when it is certain that tubercles are very rare in carnivorous animals? It is extremely probable that many patients afflicted with incurable diseases would be benefited by more liberal diet than they are usually allowed. Consumptive and cancerous subjects do not always perish from inflammation; they frequently sink from repeated syncope, which results from loss of blood.

*On abstinence in diseases of the alimentary canal.* It may be presumed that no practitioner, in the present state of medical science, would propose to feed a patient labouring under the symptoms of acute and recent gastritis. But, if it be easy to shew that, in such a case, abstinence is useful, it is not so in the last stages of the disease, when the acute symptoms have vanished. The appetite of the patient is an indication which may often embolden us to give nourishment; and disgust for food is generally to be looked upon as a guide for proscribing it: but we must not be always governed by these symptoms. In many cases no desire for food exists, although the powers of the stomach are restored; and if we wait until an appetite is felt, the patient may perish from inanition, before he evinces any disposition to eat. I have known many patients, who, after gastritic inflammation, had been kept for fifteen days upon a most rigid system of abstinence. Every symptom had disappeared, but no appetite returned. This state continued for many days, and only ceased when some light nourishment was taken, which much more rapidly restored the appetite than bitter infusions. A disgust for food is frequently the result of a thick covering upon the tongue. It has already been shewn that this is only the effect of the desiccation of the fluids which exist in the mouth.* This coat upon the tongue becomes acid, and even putrefies, by contact with the air, and communicates its odour and taste to the food. So long as it exists, and adheres firmly to the tongue, no appetite is felt, but there is a disgust for food. Neither broths nor soups will detach it: it can only be removed by the mastication of some solid food; and frequently a small quantity of bread, chewed for a long time, will restore the natural taste of the patient. Hence, then, want of appetite, loathing of food, and a foul tongue, are not always sufficient symptoms to justify our depriving the patient of nourishment. But there are other circumstances which may lead the practitioner into error. Pain in the epigastrium, which had existed a long time, in spite of abstinence, has suddenly ceased in several cases which I have observed, when the patients took food. Heat in the præcordial region, under the same circumstances, has sometimes appeared to yield to the same means. Watery fluids will

* On the Tongue, considered in reference to Diagnosis.—Journ. Hebdom. t. v. p. 305; and London Med. and Phys. Journal, May 1830, p. 454.

frequently be rejected from the stomach by vomiting, when more nourishing food will be retained.

We must not be apprehensive of the slight reaction which may arise from the first impression produced by food after long abstinence: it will soon cease; and, even if a little indigestion occur, no danger need be feared, because the quantity of food at first taken will, of course, be very small.

The softening of the mucous membrane of the stomach, after long-continued diseases, is, perhaps, frequently the result of rigorous abstinence, to which the patient has been submitted. Long-continued deprivation of food causes gastritis. The symptoms which have existed in persons who have died from inanition, and the organic alterations presented after death, prove this fact. The symptoms are usually chronic. We must be very cautious, however, how we begin to nourish persons who have long been without food. Chronic hepatitis does not appear to me to require very meagre diet: it is not by depriving the intestines of the presence of aliment, that we shall cause the biliary fluid to flow more freely into them. In organic lesions of the digestive canal, in cancerous diseases, for example, there is much to fear from too sparing a diet. If we have reason to suspect, from the symptoms, that some incomplete obstruction exists to the passage of the chyme, we may increase the contraction, by preventing food from passing through the already narrowed opening.

*Of abstinence in diseases of the Brain, and its membranes,* The nervous system suffers in various ways. Too much blood may be determined to it, and yet no symptoms may arise. Deficiency of blood in the head produces disturbance of the brain, which strongly resembles encephalic congestion or irritation. We can frequently determine that the brain is suffering, without being certain whether the affection is idiopathic or sympathetic, or whether it is the result of excitement of the cerebral mass, from lesion of its membranes, or of an excitement of the nervous substance, from visceral inflammation. Pain in the head, delirium, convulsions, and that train of symptoms termed ataxic, often happen in cases of gastro-enteritis, when the meninges of the brain are not inflamed. In hysteria, in pure inflammation of the membranes of the brain, in simple nervous excitement, and in very irritable subjects, after severe hemorrhage, when the brain is insufficiently excited from diminution of blood in its vessels, and also from abstinence, the same symptoms will arise. It is the latter fact alone which falls within the circle of the present inquiry.

Prolonged abstinence may disturb the cerebral functions in two ways. The stomach may suffer from hunger, and re-act upon the nervous system, or the natural expenditure of blood not being repaired by an adequate formation of that fluid, its quantity is much diminished, and it is deteriorated in quality. From both these causes the most serious symptoms may arise, and, if the cause be not recognised, the result may be fatal. Complete deprivation of food, for a few hours, is sufficient, in certain persons, to produce that capricious affection to which has been given the name of *hemicrania,* or *megrim.* I do not believe that the seat of this essentially nervous disease is commonly in the brain: the eye is more frequently its *point de départ;* but it is certain that a series of consecutive symptoms occur, which indicate suffering of the brain, and the parts connected with it. Severe pain in one organ of sense, extending towards the cranium, hallucination of the sight, consecutive vomiting, sometimes a painful oscillation over one half of the body, which passes from the fingers or the toes towards the trunk, are the principal symptoms which arise, and which leave no doubt as to the disturbance of the nervous system; and still these symptoms, serious as they are, are sometimes produced by a trifling delay beyond the ordinary hour of taking food. Many persons become delirious from long-continued abstinence: so true is this assertion, that it has become a popular proverb. The quantity of blood, and its stimulating power, being diminished, are sufficient to give rise to these symptoms. In several cases, where patients became delirious from rigorous abstinence, I have known the cerebral symptoms instantaneously calmed by the employment of food. The following example well illustrates this fact: A man, thirty years of age, had been subject to hemicrania, whenever he suffered hunger for some time. He was naturally irritable, and presented all those organic indications which are assigned to the nervous temperament; for a long time he had been a prey to much mental anxiety, and he was also a determined and persevering student. He was attacked with gastro-intestinal symptoms, which were quickly complicated with a very serious cerebral affection; violent headach, frightful dreams, furious delirium, tetanic stiffness of the limbs, &c. There was every reason to presume that he had inflammation of the meninges of the brain. From the kind attention of several able physicians, who saw him, these symptoms were removed in a few days. General and local bleedings, derivatives, and cold affusions, were very serviceable. He improved for several days: there was but little delirium whilst he was perfectly awake, but the moment his eyes were closed, and he endeavoured to repose himself from his fatigues, the most dreadful hallucinations haunted his imagination, which was powerfully excited by severe suffering. Eight days had elapsed since the commencement of the disease. The original symptoms were relieved in three days, but then these hallucinations became more and more terrible. The strictest diet was ordered: the patient, reduced to a state of extreme weakness, felt an insupportable sensation of hunger, and was now attacked with his ordinary hemicrania. He was convinced that the nightly delirium arose from his rigorous abstinence, and he took some very light nourishment. In four hours he slept tranquilly, and his ideas no longer wandered. He gradually increased his diet, the delirium did not reappear, and he was soon convalescent.

In typhoid fevers, cerebral symptoms are not unfrequently the result of the want of a proper portion of food; and as to those nervous symptoms which follow great surgical operations, we must always remember that the patient has often lost a large quantity of blood, is frequently weakened by long suffering and privations of every kind.

In young children, especially, we must be very cautious of abstinence from food. This truth, which has been transmitted to us from the time of Hippocrates, is of much practical importance. The most serious cerebral symptoms are very frequently produced in them from prolonged low diet, and most of the symptoms of inflammation of the membranes of the brain may arise in consequence of privation of food. A child, three years old, was attacked with severe diarrhœa, which yielded to the application of leeches and strict diet. The latter was continued even after the cessation of the symptoms for which it had been advised: fever had disappeared, and every circumstance seemed to indicate rapid recovery. For ten days the little patient had taken nothing more than gum water in small quantities. Slight convulsive twitchings took place, with delirium and headach; the eyes were thrown upwards spasmodically; profound coma supervened, the child, however, rousing every now and then with piercing shrieks. This state lasted two days, and no food was allowed, from the fear of augmenting the cerebral symptoms. The skin became cold, face pale, with occasional flushing of the cheeks. Quinine, given in enemata, did no good, and the death of the child appeared certain. After twelve days' abstinence, and when no hope seemed to remain, diluted milk was given by spoonsful every half hour: the screaming and convulsions were now less frequent. The quantity of milk was increased, and the cerebral symptoms ceased: in the morning, the child was much better, and even a little cheerful. Pure milk was now given without injury: the coma, delirium, and headach disappeared. The quantity of food was gradually increased, and the cure was as rapid as it was unexpected. A few days afterwards, a child, four years old, presented the same symptoms, and they yielded to similar treatment.

When cerebral symptoms occur in young children, after copious evacuations, we should be on our guard against the disturbance, which is more likely to arise from want of blood than from sanguineous congestion. In such cases I have known convulsions occur directly the head was raised, and cease the moment it was placed upon a level with the body. This fact was strongly exemplified in the case of a child, five years old: after death, no marks of cerebral inflammation were detected; the vessels were nearly empty. The convulsions which occurred in this case were precisely similar to those which arise from inflammation of the membranes of the brain, and they did not differ from those convulsive movements which sometimes immediately follow loss of blood in adults.

Such are the principal facts which have long attracted my attention. The practice of a large hospital, dedicated to old persons, has confirmed my ideas. In such subjects, loss of blood produces but a momentary weakness, provided a due degree of nourishment is allowed. The regimen of a patient is of the utmost consequence: it is one of our leading means of cure; it must vary according to particular circumstances; but there are certainly as many cases in which liberal diet will be advantageous, as there are in which a diminution, or even total abstinence, from food is necessary. Experience must teach the physician which plan he is called upon to adopt; but let us not lose sight of the undoubted truth, that a sick man cannot live long without food, any more than a healthy one.

